# *Sideritis scardica* Griseb. Essential Oil as Potential Antimicrobial Agents—A Study of Their Composition and Activity

**DOI:** 10.3390/molecules31091515

**Published:** 2026-05-02

**Authors:** Rafał Papliński, Agnieszka Grzegorczyk, Renata Nurzyńska-Wierdak, Magdalena Walasek-Janusz

**Affiliations:** 1 Department of Vegetable and Herb Crops, Faculty of Horticulture and Landscape Architecture, University of Life Sciences in Lublin, 54 Doświadczalna Street, 20-280 Lublin, Poland; rafal.paplinski@up.lublin.pl (R.P.);; 2 Department of Pharmaceutical Microbiology, Faculty of Pharmacy, Medical University of Lublin, 1 Chodzki Street, 20-093 Lublin, Poland

**Keywords:** mountain tea, secondary metabolites, diversity, GC-MS analysis

## Abstract

The essential oil (EO) of *Sideritis* L. has attracted great interest due to its pharmacological activities. At the same time, there is significant variability within the type, related, among other things, to the origin of the raw material. The aim of this work was to study the EO chemical composition of *Sideritis scardica* Griseb. from Bulgaria and Türkiye. The plant material (air-dried above-ground parts) was purchased from herbal and medical stores in Lublin, Poland. The crushed raw material was used for distillation of the EO. Distillation was performed in a Clevenger apparatus. The EO content was expressed in ml per 100 g of air-dried herb. Analysis of the qualitative and quantitative composition of the obtained EO was performed using gas chromatography coupled with a mass spectrometer (450-GC + 240-MS). The antimicrobial activity of the *S. scardica* EO was evaluated using the broth microdilution method in accordance with the guidelines of the European Committee on Antimicrobial Susceptibility Testing (EUCAST) guidelines. We have demonstrated that the chemical composition and biological activity of sideritis EO depend on the origin of the raw material. Our results indicate that *S. scardica* EO can be considered a promising antimicrobial agent.

## 1. Introduction

The genus *Sideritis* L. (Lamiaceae) comprises over 150 species widely distributed in the Mediterranean region, including the Canary Islands and Madeira. The above-ground parts of these plants (herb), known as mountain tea, have been and continue to be traditionally used as aromatic herbal teas, flavourings and fragrances, as well as anti-inflammatory, anti-ulcer, antimicrobial, analgesic, antioxidant, antispasmodic, anticonvulsant, analgesic and carminative agents [1,2,3]. Aromatic herbs are an essential component of the Mediterranean diet, one of the healthiest in the world and recognised as part of humanity’s intangible cultural heritage. Mountain tea, made from dried flowers and leaves of the sideritis plant, is a traditional herbal tea in Greece, the Balkans, the Iberian Peninsula and Türkiye. In Greece and the Balkans, the most popular species of sideritis for making mountain tea are *S. raeseri* Boiss. & Heldr and *S. scardica* Griseb. *S. scardica*, also known as Olympus tea, is found in rocky areas and alpine regions of Northern Greece, up to the Olympus mountain range and Mount Pelion. It is a species endemic to the Balkan Peninsula, known in south-eastern Europe for its cultivation and traditional uses (relieving colds, calming purposes) [2,4,5]. Because of its high popularity and excessive, unregulated wild harvesting, *S. scardica* is regarded as an endangered species; it is listed in the Red Book of Bulgaria and is legally protected [6]. In Northern Greece, *S. scardica* predominates in habitats rich in flowering herbaceous plants, whereas the presence of grasses, sedges, rushes, and shrubs restricts its distribution. This species is characterised by a high coefficient of variation, ranging from 12.2 to 29.2% to 13.3–43.1% for inflorescence and leaf characteristics [7].

Seskiterpenes, diterpenes, triterpenes, sterols, flavones, lignans, coumarins and other aromatic compounds have been isolated from nine Canarian *Sideritis* species [8]. These substances are found in almost all sideritis species, and are the main compounds responsible for their pharmacological effects [3,9]. Sideritis essential oil (EO) consists of many dominant compounds, mainly monoterpenes and sesquiterpenes. Trendafilova et al. [10] demonstrated significant variability in the chemical composition of the EO of six native populations of *S. scardica* from Bulgaria and a correlation between the EO profile and the ecological conditions of the habitat. Kloukina et al. [11] presented the EO composition of *S. scardica* herb from organic farms in various locations in Kozani (central Greece). The *S. scardica* herb from Metamorfosis Kozani and *S. scardica* herb from Chromio Kozani contained 0.18% and 0.12% EO, respectively. The main fraction of the tested *S. scardica* EO was monoterpene hydrocarbons. Some components (α-pinene, β-pinene, cis-caryophyllene, bicyclogermacrene and germacrene D) were found in varying amounts in both *S. scardica* EO samples. Kostadinova et al. [12], studying EOs of *S. scardica* and *S. raeseri* growing in Bulgaria and Macedonia, showed that *S. scardica* EO from different locations differed significantly. The observed variability in the chemical composition of *S. scardica* EO of different geographical origins may be the result of different ecological conditions, as well as the tendency of some *Sideritis* species to hybridise. Similarly, Kaparakou et al. [13] report that EOs obtained from *S. scardica* and *S. raeseri* originating from three different regions of Greece and *S. syriaca* originating from three different locations in Crete showed high variability in chemical composition, which can be explained by environmental variability.

*S. scardica* is of great ethnopharmacological importance and is used in modern phytotherapy [14]. The European Medicines Agency (EMA) has recognised *Sideritis* spp. as medicinal plants in traditional uses, and infusions of *S. scardiaca* Griseb., *S. clandestina* (Bory and Chaub.) Hayek, *S. raeseri* Boiss. and Heldr., and *S. syriaca* L. as a remedy for colds, to relieve coughs, and also for mild gastrointestinal disorders [14,15]. Findings reported by Behrendt et al. [4] indicate that a six-week supplementation with *S. scardica* extract combined with B vitamins may alleviate the effects of mental stress, enhance stress resilience, and improve both cognitive performance and visual attention under acute stress conditions. According to the authors, the results of the experiment may be relevant for people solving cognitive tasks in conditions of conflict and/or noise (e.g., open-plan offices or driving a car). Heiner et al. [16] report that extracts from *S. scardica* containing the phenyl-ethanoid glycoside acetozyd exhibit activity against Alzheimer’s disease, indicating potential applications in the treatment or prevention of neurodegenerative diseases. EOs from *Sideritis* L. are of great interest not only for their pharmacological effects, but also for their potential applications in the cosmetics and perfume industries. These substances were tested for their antimicrobial, antioxidant, anti-inflammatory and anti-proliferative effects [13]. Ultimately, scientific research results indicate significant variability in the chemical composition and biological activity of EOs from sideritis and confirm the need for in-depth research on EOs obtained from sideritis plants originating from different locations [1,2,3,4,5,6,7,8,9,10,11,12,13,17]. Koutsaviti et al. [18] demonstrated that EOs from sideritis species exhibit significant activity against certain strains of microorganisms, with EO from *S. lanata* showing MIC values for *Staphylococcus aureus* and *Micrococcus luteus* comparable to those of reference antibiotics. The objective of this study was to analyse and compare the chemical composition and antimicrobial properties of the essential oil (EO) derived from one of the most popular mountain tea species, *S. scardica*, using plant material obtained from Bulgaria and Türkiye.

## 2. Results

### 2.1. GC-MS Analysis of S. scardica Essential Oil and Distillation of Essential Oil (EO)

Significant variation in EO content was observed in the plant material studied (Table 1). The raw material from Bulgaria (SEO 1) contained less essential oil than the Türkiye raw material (SEO 2) (0.08 and 0.76 mL·100 g^−1^, respectively).

**Figure 1:**
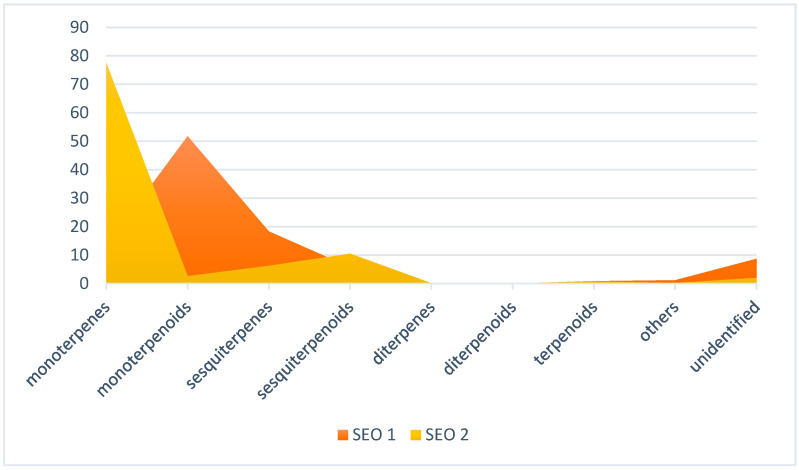
Groups of compounds found in sideritis EOs (%).

**Figure 2:**
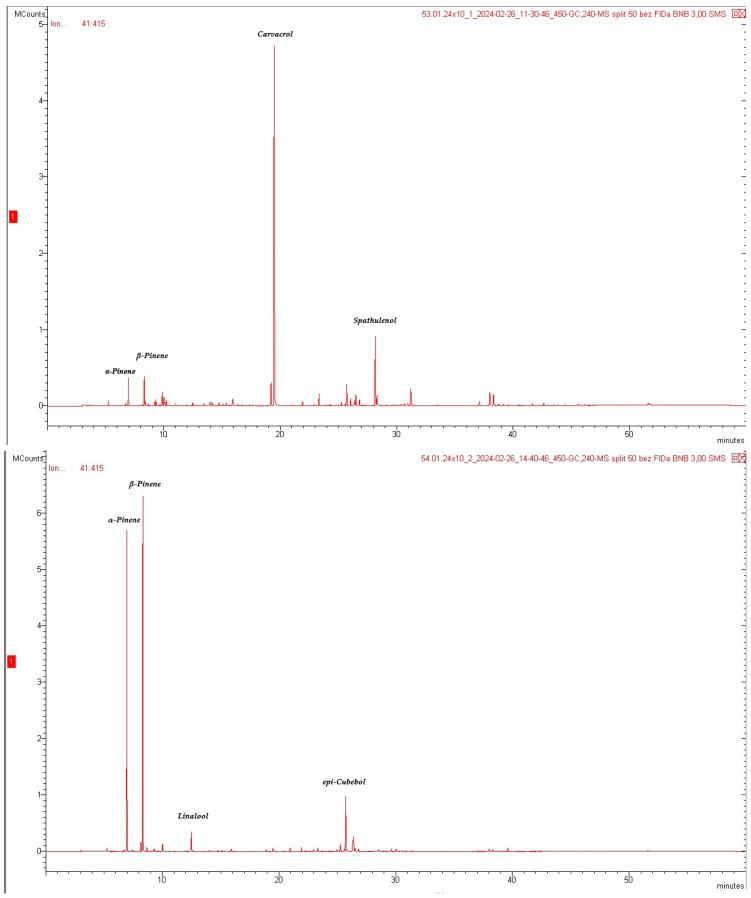
GC-MS chromatograms of EOs obtained from sideritis herb (from the top: SEO 1 and SEO 2).

The chemical composition of the tested EOs varied both in terms of the number of components and their concentration (Table 2, Figure 1 and Figure 2).

Terpenes and their terpenoid derivatives are one of the most numerous groups of secondary metabolites. These compounds exhibit great diversity in terms of chemical structure and biological activity. For this reason, the classification (Figure 1) was carried out taking into account the above groups of compounds and their derivatives.

Monoterpenes (13.26 and 77.49%), monoterpenoids (51.76 and 2.62%), sesquiterpenes (18.33 and 6.22%), sesquiterpenoids (5.72 and 10.51%), diterpenes (0.20 and 0.10%), diterpenoids (0.00 and 0.08%), terpenoids (0.87 and 0.78%), others (1.17 and 0.21%), unidentified compounds (8.69 and 1.99%). Raw material from: Bulgaria (SEO 1) and Türkiye (SEO 2).

In the EO obtained from raw material originating in Bulgaria (SEO 1), 103 compounds were identified, of which 25 were not identified, accounting for 99.97% of all components, with monoterpene compounds accounting for 59.99% and sesquiterpene compounds accounting for 18.33%. The dominant compound in SEO 1 was carvacrol (46.73%), a natural monoterpene derivative of cymene (Table 2; Figure 1), followed by spathulenol (7.93%), β-pinene (3.01%), thymol (2.94%), α-pinene (2.67%), bicyclogermacrene (2.49%), and α-bisabolol (2.42%). In the EO obtained from raw material originating in Türkiye (SEO 2), 68 compounds were detected, of which 13 were not identified, accounting for 99.82% of the total composition. The dominant groups of compounds were monoterpenes (77.49%) and sesquiterpenoids (10.51%). The main compounds were β-pinene and α-pinene, with contents of 40.11% and 32.16%, respectively, followed by epi-cubenol (7.35%), linalool (2.26%) and δ-cadinene (2.32%) (Figure S1).

### 2.2. The Antimicrobial Activity of the Sideritis EOs

**Figure 3:**
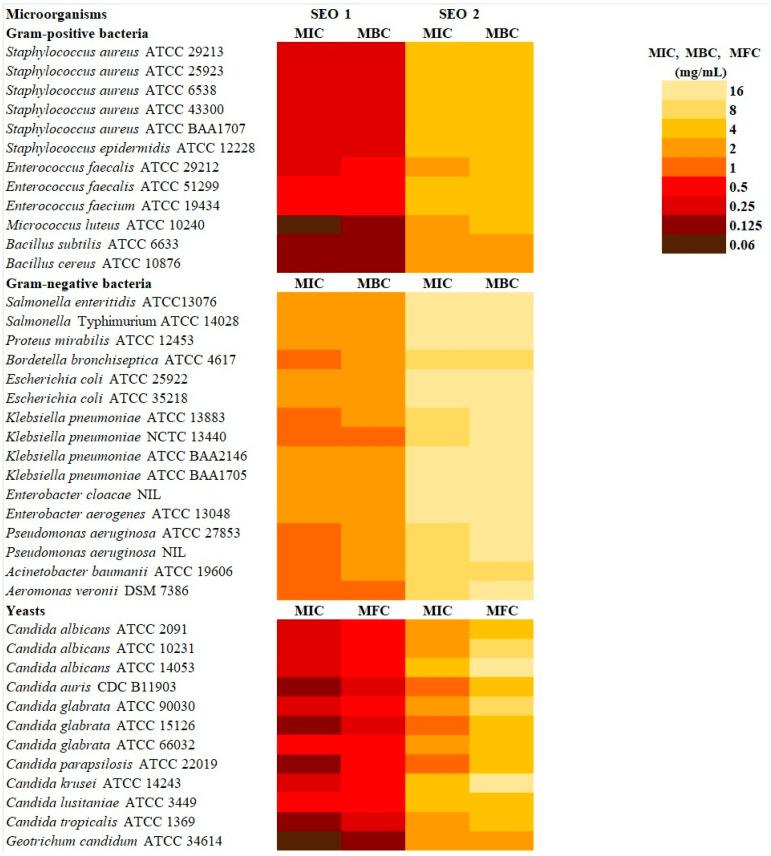
Antimicrobial activity (MIC, MBC, and MFC doses) of sideritis EOs against tested microorganisms.

The antimicrobial activity of two sideritis EOs, SEO 1 and SEO 2, was assessed against a panel of Gram-positive and Gram-negative bacteria, as well as yeast strains. The results of the minimum inhibitory concentration (MIC), minimum bactericidal concentration (MBC), and minimum fungicidal concentration (MFC) tests are summarised in Figure 3.

Both EOs (SEO 1 and SEO 2) demonstrated distinct antimicrobial profiles against a panel of clinically relevant bacteria and yeasts. Strikingly, SEO1 consistently outperformed SEO2, demonstrating significantly lower MIC, MBC, and MFC values against both Gram-positive and Gram-negative bacteria, as well as fungal pathogens.

Among Gram-positive bacteria, SEO1 exhibited MIC values ranging from 0.06 to 0.5 mg·mL^−1^, with strong activity observed against *Micrococcus luteus* (MIC = 0.06 mg·mL^−1^, MBC = 0.125 mg·mL^−1^) and *Bacillus* spp. (MIC and MBC = 0.125 mg·mL^−1^), and very good activity against *Staphylococcus* spp. and *Enterococcus* spp. strains (MIC = 0.25–0.5 mg·mL^−1^). In contrast, SEO2 exhibited significantly weaker inhibition, with MIC values of 2–4 mg·mL^−1^ against the same strains.

Although Gram-negative bacteria are typically less susceptible to EOs due to their protective outer membrane, SEO1 retained good activity, with MIC values ranging from 1 to 2 mg·mL^−1^, while SEO2 required 8 to 16 mg·mL^−1^ to achieve comparable effects. Remarkably, *Bordetella bronchiseptica*, *Klebsiella pneumoniae* (NCTC 13440, ATCC 13883), *Pseudomonas aeruginosa* (ATCC 27853, NIL), *Acinetobacter baumannii*, and *Aeromonas veronii* demonstrated good susceptibility (MIC = 1 mg·mL^−1^), underscoring the broad potential of this essential oil.

A similar trend was observed in antifungal assays. SEO1 inhibited the growth of all *Candida* spp. and *Geotrichum candidum* at MICs ranging from 0.06 to 0.5 mg·mL^−1^, whereas SEO2 showed notably weaker activity (MIC = 1–4 mg·mL^−1^). *G. candidum* and *C. tropicalis*, *C. parapsilosis*, *C. glabrata* ATCC 15126, and *C. auris* were the most susceptible to SEO1, with MIC values of 0.06 mg·mL^−1^ and 0.125 mg·mL^−1^, respectively.

Importantly, the MBC/MIC and MFC/MIC ratios for both oils were ≤4, indicating that they have bactericidal and fungicidal effects, rather than merely inhibitory ones.

## 3. Discussion

The genus *Sideritis* L., which includes many plant species found in temperate and tropical regions of the northern hemisphere, is characterised by high variability in chemical composition. Some species have a high essential oil content and are used as flavourings and medicinal agents [14,19,20]. Our studies showed a high average content of *S. scardica* EO (an average of 0.42% for both raw materials studied), higher than that reported by most authors [11,13]. The Bulgarian and Türkiye raw materials we analysed showed notable differences in both essential oil content and its chemical composition. The Türkiye raw material was distinguished by a higher essential oil content (0.76%) than that determined in the Bulgarian raw material (0.08%). Özcan et al. [20] also found a similarly high essential oil content, although with some variation in this respect for the raw materials *S. bilgerana*, *S. tmolea* and *S. congesta* (0.26%, 0.33% and 0.83% respectively) sourced from different regions of Turkey. Kaparakou et al. [13] demonstrated the low EO content of *S. scardica* (0.02–0.05%) originating from three different regions of Greece (Central, Southern, and Northern) and its diverse chemical composition. The common components of the EOs were: 1-octen-3-ol; benzaldehyde; linalool; p-mentha-1,5-dien-8-ol; trans-pinocarveol; α-terpineol; myrtenol; verbenone and eugenol. In turn, Kloukina et al. [11] report higher yields of EOs from the aerial parts of *S. scardica* grown in different regions of Greece (0.18% and 0.12%). Interestingly, two different samples of *S. scardica* showed similar chemical composition with slight differences in the amounts of individual volatile components. The main fraction was monoterpene hydrocarbons. Overall, the variability in essential oil content of *Sideritis* is linked to genetic and ontogenetic differences, as well as growing conditions and post-harvest practices.

The dominant group of EOs in *S. scardica* may be mono- or sesquiterpene hydrocarbons, which are related to environmental conditions [11,21]. Trendafilova et al. [10] identified the following as the dominant components of *S. scardica* EO: α-pinene (4.4–25.1%), β-pinene (2.8–18.0%), oct-1-en-3-ol (2.3–8.0%), phenylacetic aldehyde (0.5–9.5%), β-bisabolene (1.3–11.0%), benzyl benzoate (1.1–14.3%) and m-camphorene (0.3–12.4%). All samples were characterised by low content of oxidised mono- and sesquiterpenes (≤1.6 and 2.3%, respectively). Qazimi et al. [2] identified 30–43 components of *S. scardica* and *S. raeseri* EOs, noting similarities in chemical composition. The main components of *S. scardica* EO were identified as: β-pinene (8.2–40.6%), α-pinene (4.2–23.3%) and limonene + β-phellandrene (3.5–16.7%), 1-octen-3-ol (6.2–29.8%), linalool (1.6–3.1) and trans-caryophyllene (6.7–15.6%), followed by α-copaene, germacrene D and bicyclogermacrene. Kostadinova et al. [12] studied the EO of wild-growing *S. scrdica* plants in Bulgaria and Macedonia: The EOs differed significantly: the Macedonian sample was dominated by a-cadinol (20%), while in the EO from the Bulgarian samples, the main components were diterpene compounds and octadecenol (over 20%). The results of these authors contrast with our results and with the results of studies on *S. scardica* growing in Greece, where monoterpene hydrocarbons and/or phenolic compounds, thymol and carvacrol, were identified as the most abundant components of the essential oil. No phenolic compounds, menthol, nerol or geraniol, the main components of essential oil from *S. scardica* originating from Yugoslavia [22], were found in the samples described by Kostadinova et al. [12]. The observed variability in the quality of the essential oil composition of *S. scardica* from different geographical origins may be the result of environmental variability.

This study indicates a correlation between the chemical composition of *S. scardica* essential oils and their antimicrobial activity. Our previous studies [23] demonstrated a varied polyphenol profile, as well as antioxidant and antimicrobial activity, in the *S. scardica* raw material examined here. The Türkiye raw material contained more polyphenolic compounds than the Bulgarian raw material, although both raw materials exhibited comparable antioxidant activity. The Türkiye sample exhibited greater therapeutic potential than the Bulgarian sample, particularly against Gram-positive pathogens and *Candida* species, acting mainly through bactericidal and fungistatic mechanisms. In this study, a clear difference in susceptibility was observed between Gram-positive and Gram-negative bacteria, resulting from the different chemical composition of these two essential oils. The essential oil obtained from Bulgarian plant material (SEO 1), characterised by a high content of phenolic monoterpenes, especially carvacrol (46.73%), exhibited significantly stronger antimicrobial activity than the essential oil from Türkiye (SEO 2), which was dominated by monoterpene hydrocarbons, mainly α-pinene and β-pinene. The higher antimicrobial efficacy of SEO 1 has been described by other authors, who noted that phenolic monoterpenes, such as carvacrol and thymol, are among the most potent antimicrobial components of essential oils [24]. It has also been proven that these compounds destroy microbial cell membranes, increasing their permeability and leading to the leakage of intracellular components, which ultimately results in cell death [25]. In contrast, monoterpene hydrocarbons, such as α-pinene and β-pinene, generally exhibit weaker antimicrobial activity, as observed for SEO 2 [26]. Thus, the significantly lower antimicrobial activity of SEO 2 can be attributed to the predominance of monoterpene hydrocarbons and the absence of high levels of phenolic compounds [27]. SEO 1 exhibited strong activity against Gram-positive bacteria, which is consistent with previous studies showing higher susceptibility of Gram-positive bacteria to essential oils [28]. This is primarily due to the absence of an outer membrane, which facilitates the penetration of hydrophobic compounds. In contrast, Gram-negative bacteria were less susceptible, as they possess an outer membrane rich in lipopolysaccharides, which acts as a barrier to hydrophobic molecules. The observed activity against strains such as *Pseudomonas aeruginosa* and *Acinetobacter baumannii* suggests that oils rich in carvacrol can overcome this barrier, likely by destabilising the membrane [24]. The potent activity of SOE 1 against *Staphylococcus aureus* (including MRSA strains) and *Enterococcus* spp. suggests that this compound has potential for therapeutic or preservative applications, particularly in cases where there is concern about resistance to conventional antibiotics.

The antifungal activity of essential oils from *S. scardica* that we observed confirms the importance of phenolic compounds as antimicrobial agents. SEO 1, which contains a high concentration of carvacrol, exhibited strong activity against *Candida* spp., which is consistent with reports by other authors indicating that carvacrol and thymol interact with ergosterol in fungal cell membranes, leading to increased permeability and growth inhibition [29]. Carvacrol reduced the adhesion of *C. auris* to surfaces and its production of proteases [30]; it also exhibited synergistic or additive effects when used in combination with antifungal drugs such as fluconazole, amphotericin B, nystatin and caspofungin [31]. Furthermore, it was observed that both essential oils exhibited bactericidal and fungicidal activity, as evidenced by MBC/MIC and MFC/MIC values ≤ 4. According to EUCAST criteria, this indicates bactericidal activity rather than mere growth inhibition. The oil’s effectiveness may be due to synergistic interactions among its components, which can modulate antimicrobial activity [27].

## 4. Materials and Methods

### 4.1. Plant Material

The test material consisted of sideritis herb (above-ground parts of plants) originating from Bulgaria (SEO 1) and Türkiye (SEO 2), appropriately crushed and dried. The plant material was purchased in herbal and medical shops in Lublin (Poland). The samples are listed in Table 3. The crushed raw material was used for hydrodistillation of essential oil using a Clevenger-type apparatus.

### 4.2. Distillation of EO from Sideritis Herb

The distillation of EO was carried out using sideritis herb sourced from Bulgaria (SEO 1) and Türkiye (SEO 2). For distillation, 20 g of raw material was weighed and placed in a round-bottom flask, then 450 mL of distilled water was added. The whole mixture was placed in a heating mantle and heated for 3 h from the moment the water reached boiling point. The distillation was carried out in a Clevenger apparatus (Chemland, Stargard, Poland), in three repetitions. After the required time had elapsed, the heating mantle was switched off and after 15 min the EO obtained was brought to a micro-scale and the result was read. The EO content was expressed in ml per 100 g of dried herb (Table 2). The obtained EO was stored at 4 °C until GC-MS analysis and microbiological testing were performed.

### 4.3. GC-MS Analysis of EOs from Sideritis spp.

The semi-quantitative analysis of the obtained EOs was performed using gas chromatography coupled with mass spectrometry (450-GC + 240-MS), using a Varian apparatus (Varian Medical Systems, Warszawa, Poland). The EO sample for direct testing was diluted 1000 times in hexane. Then, 1 µL of the diluted sample was injected for analysis, while the injector temperature was 250 °C, with a split of 50. Helium was used as the carrier gas at a constant flow rate of 1.0 mL/min and a VF-5 ms column. The temperature in the column furnace was 50 °C and was maintained for 1 min, then increased to 240 °C at a rate of 4 °C/min, and the temperature of 240 °C was maintained for 10 min. The detector was a mass spectrometer with an ion trap with electron ionisation (EI). Full scanning was used in the range of 41–415 m/z, and the scanning speed was 0.8 s per scan. The total analysis time was 60 min. Compounds were identified based on mass spectra, according to Adams [32] and the NIST Mass Spectra Library [33]. Retention indices of the detected compounds were determined on a VF-5 ms column, using a series of n-alkanes (C7-C40 Saturated Alkanes Standard, Product no.: 49452-U Supelco; Merck, Darmstadt, Germany ) [34].

### 4.4. Microbiological Analyses

The antimicrobial activity of *S. scardica* essential oils was evaluated using the broth microdilution method in accordance with the guidelines of the European Committee on Antimicrobial Susceptibility Testing (EUCAST) of the European Society of Clinical Microbiology and Infectious Diseases (ESCMID): determination of minimum inhibitory concentrations (MICs) of antibacterial agents by broth dilution [35], as previously described [36]. Serial dilutions of the essential oils were prepared to achieve final concentrations of 16, 8, 4, 2, 1, 0.5, 0.25, 0.125, 0.06, and 0.03 mg·mL^−1^. This method was employed to determine the minimum inhibitory concentration (MIC), minimum bactericidal concentration (MBC), or minimum fungicidal concentration (MFC) of each essential oil against twenty-three microbial strains under in vitro conditions.

The antimicrobial efficacy of the *S. scardica* essential oils was assessed against reference strains obtained from the American Type Culture Collection (ATCC), comprising twelve Gram-positive bacteria strains (*Staphylococcus aureus* ATCC 29213, ATCC 6538P, ATCC 25923—methicillin-sensitive; *Staphylococcus aureus* ATCC 43300, ATCC BAA1707—methicillin-resistant; *Staphylococcus epidermidis* ATCC 12228; *Enterococcus faecalis* ATCC 29212, ATCC 51299; *Enterococcus faecium* ATCC 19434; *Micrococcus luteus* ATCC 10240; *Bacillus subtilis* ATCC 6633; *Bacillus cereus* ATCC 10876), eleven Gram-negative bacteria strains (*Salmonella enteritidis* ATCC13076; *Salmonella* Typhimurium ATCC 14028; *Proteus mirabilis* ATCC 12453; *Bordetella bronchiseptica* ATCC 4617; *Escherichia coli* ATCC 25922 and ATCC 35218; *Klebsiella pneumoniae* ATCC 13883 and ATCC BAA2146; *Enterobacter aerogenes* ATCC 13048; *Pseudomonas aeruginosa* ATCC 27853; *Acinetobacter baumanii* ATCC 19606), and twelve yeast strains (*Candida albicans* ATCC 2091, ATCC 10231, 14053; *Candida auris* CDC B11903; *Candida glabrata* ATCC 90030, ATCC 15126, *Candida parapsilosis* ATCC 22019; *Candida krusei* ATCC 14243; *Candida lusitaniae* ATCC 34449; *Candida tropicalis* ATCC 1369; *Geotrichum candidum* ATCC 34614; *Candida glabrata* ATCC 66032).

All assays were performed in triplicate. In each experiment, appropriate controls were included. Growth controls (inoculated medium without essential oil) and sterility controls (non-inoculated medium) were used to verify microbial viability and medium sterility, respectively. Additionally, control wells containing culture medium with essential oils and their serial dilutions, but without inoculum, were included to exclude any interference of the tested substances with the assay. Standard antimicrobial agents were used as positive controls: fluconazole (0.06–16 µg·mL^−1^) for yeasts, ciprofloxacin (0.015–16 µg·mL^−1^) for Gram-negative bacteria, and vancomycin (0.06–16 µg·mL^−1^) for Gram-positive bacteria. The obtained MIC values for reference strains were within the expected ranges (e.g., 1 µg·mL^−1^ for fluconazole against *Candida albicans* ATCC 10231, 1 µg·mL^−1^ for vancomycin against *Staphylococcus aureus* ATCC 29213, and 0.015 µg·mL^−1^ for ciprofloxacin against *Escherichia coli* ATCC 25922), confirming the validity of the method.

### 4.5. Statistical Analysis

The obtained results are presented as means and were analysed statistically by ANOVA according to a completely randomised design, and the average values were compared using the HSD Tukey’s test at the probability level α = 0.05. The statistical analysis of the results was carried out with the Statistica 13.3 PL package (Statsoft, Inc., Tulsa, OK, USA).

## 5. Conclusions

The *S. scardica* Griseb. raw material was characterised by a variable essential oil content, depending on its origin. The Türkiye plant material was distinguished by a 10-fold higher essential oil content compared to the Bulgarian material. *S. scardica* essential oil, which exhibits a monoterpene-sesquiterpene chemotype, displays significant antioxidant and antimicrobial activity, depending on its origin (chemical composition). The presence of carvacrol in the essential oil (Bulgarian source), known for its antimicrobial activity, may explain its significantly greater activity against Gram-positive and Gram-negative bacteria as well as yeasts, than the second one. Regardless of the origin of the raw material, *S. scardica* herb appears to be an extremely interesting subject for further research into the chemical profile of its essential oil, its biological activity and its potential therapeutic applications. The complex chemical composition of herbal materials offers numerous opportunities for chemical and biological analysis. It should be emphasised that, when establishing quality control standards for *Sideritis* raw material, the assessment should not be limited to essential oils, but should also include non-volatile components.

## Figures and Tables

**Table 1:** The EO content in the tested sideritis herb (%).

Plant Material	Average EO Content
SEO 1	0.08 a
SEO 2	0.76 b
Mean	0.42

Data related to a given compound in columns marked with the same letter do not differ significantly from each other by the Tukey test at 5% of probability.

**Table 2:** Chemical composition of sideritis EOs (%).

No	Compound	RT	RI	SEO 1	SEO 2
1	(2E)-Hexenal	4.90	853	0.54	-
2	Tricyclene	6.64	923	-	0.63
3	α-Thujene	6.72	926	0.22	0.17
4	α-Pinene	6.95	933	2.67	32.16
5	Camphene	7.46	950	0.51	0.22
6	Thuja-2,4(10)-diene	7.58	954	0.62	-
7	Verbenene	8.13	972	tr	-
8	Sabinene	8.16	973	0.12	0.97
9	β-Pinene	8.33	978	3.62	40.11
10	1-Octen-3-ol	8.40	981	0.51	0.12
11	Myrcene	8.67	990	0.24	0.42
12	α-Phellandrene	9.25	1008	0.37	0.18
13	δ-3-Carene	9.33	1010	0.64	0.35
14	α-Terpinene	9.60	1018	0.12	-
15	m-Cymene	9.70	1021	0.84	-
16	ρ-Cymene	9.87	1026	1.46	0.89
17	Limonene	10.02	1030	1.55	0.94
18	β-Phellandrene	10.08	1032	0.32	0.15
19	1,8-Cineole	10.14	1034	0.12	-
20	(Z)-β-Ocimene	10.24	1036	0.63	-
21	(E)-β-Ocimene	10.60	1047	0.94	-
22	γ-Terpinene	11.02	1059	0.25	0.82
23	Ui	11.49	1073	0.17	-
24	Terpinolene	11.97	1087	0.13	-
25	Linalool	12.49	1102	0.37	2.26
26	trans-Sabinene hydrate	12.57	1104	0.68	-
27	n-Nonanal	12.68	1107	0.66	-
28	α-Campholenal	13.48	1130	0.26	tr
29	Nopinone	13.94	1143	0.66	-
30	trans-Pinocarveol	13.98	1144	0.52	0.16
31	cis-Verbenol	14.02	1145	0.85	-
32	trans-Verbenol	14.15	1149	0.50	0.12
33	Pinocarvone	14.75	1166	0.43	0.13
34	Borneol + p-Mentha-1,5-dien-8-ol	15.09	1175	0.28	0.16
35	Terpinen-4-ol	15.37	1183	0.38	0.94
36	Cryptone	15.63	1191	0.15	-
37	α-Terpineol	15.89	1198	0.23	0.52
38	Myrtenol	15.93	1199	1.47	0.36
39	Verbenone	16.35	1212	0.16	tr.
40	Ui	16.45	1214	0.84	-
41	trans-Carveol	16.73	1222	0.14	-
42	Methyl ether thymol	17.36	1241	0.18	-
43	Carvone	17.60	1248	0.17	-
44	Bornyl acetate	18.92	1287	0.15	0.29
45	Thymol	19.21	1295	2.94	0.52
46	Carvacrol	19.48	1303	46.73	0.55
47	Hexyl tiglate	20.41	1332		0.85
48	α-Cubebene	20.96	1348	0.77	0.41
49	Ui	21.79	1374	0.13	-
50	α-Copaene	21.91	1377	0.59	0.57
51	(E)-β-Damascenone	22.06	1382	0.69	-
52	β-Bourbonene	22.18	1385	-	0.79
53	β-Cubebene	22.31	1389	-	0.17
54	Ui	22.81	1405	0.85	-
55	Ui	22.93	1409	0.24	-
56	α-Gurjunene	22.93	1409	-	0.34
57	Ui	23.04	1413	0.78	-
58	(E)-Caryophyllene	23.34	1422	1.57	0.46
59	Ui	23.94	1441	0.17	-
60	cis-Muurola-3,5-diene	24.25	1451	0.19	0.12
61	(Z)-β-Farnesene	24.33	1454	0.15	-
62	α-Humulene	24.46	1458	0.57	tr
63	allo-Aromadendrene	24.60	1463	0.57	0.57
64	trans-Cadina-1(6),4-diene	24.96	1474	0.92	0.22
65	Germacrene D	25.25	1484	0.56	1.73
66	trans-Muurola-4(14),5-diene	25.58	1494	0.25	0.29
67	Bicyclogermacrene	25.70	1498	2.49	-
68	epi-Cubebol	25.71	1498	-	7.35
69	β-Bisabolene	26.04	1510	0.97	0.71
70	Cubebol	26.32	1519	0.15	1.46
71	δ-Cadinene	26.38	1521	0.79	2.32
72	cis-Calamenene	26.49	1525	1.63	0.58
73	trans-Cadina-1,4-diene	26.81	1535	0.82	0.34
74	Ui	27.02	1542	0.73	-
75	α-Calacorene	27.08	1545	0.16	-
76	Ui	27.27	1551	0.13	-
77	(3Z)-Hexenyl benzoate	27.99	1575	0.16	-
78	Spathulenol	28.15	1581	7.93	0.13
79	Caryophyllene oxide	28.31	1586	1.62	0.96
80	Ui	28.50	1593	-	0.17
81	Ui	28.54	1594	-	0.22
82	Viridiflorol	28.67	1598	0.79	-
83	Ledol	28.97	1609	-	0.77
84	Humulene epoxide II	29.10	1614	0.12	-
85	1-epi-Cubenol	29.61	1632	0.74	0.44
86	Ui	29.76	1637	0.17	0.17
87	Cubenol	30.07	1648	0.13	0.55
88	α-Muurolol (=Torreyol)	30.15	1651	-	0.65
89	α-Bisabolol oxide B	30.37	1659	0.95	-
90	cis-Calamenen-10-ol	30.42	1661	0.27	-
91	Ui	30.49	1664	-	0.12
92	trans-Calamenen-10-ol	30.50	1664	0.13	
93	Ui	30.68	1670	0.48	0.17
94	Valeranone	30.94	1679	0.56	-
95	α-Bisabolol	31.21	1689	2.42	-
96	Ui	31.22	1689	-	0.72
97	Benzyl benzoate	33.49	1774	1.00	-
98	(Z)-Lanceol acetate	35.45	1850	0.14	0.53
99	Ui	36.94	1909	-	0.66
100	Ui	37.10	1916	0.69	0.18
101	Ui	37.67	1939	-	0.78
102	Ui	38.00	1953	2.35	-
103	Ui	38.00	1953	-	0.58
104	Ui	38.31	1965	1.71	-
105	Ui	38.31	1966	-	0.32
106	Ui	38.74	1983	0.47	0.11
107	Kaur-15-ene	39.19	2002	0.21	0.15
108	13-epi-Manool oxide	39.59	2019	0.14	0.43
109	Ui	40.46	2057	0.11	-
110	Ui	40.56	2061	0.95	-
111	Ui	40.78	2071	0.62	-
112	Ui	41.65	2109	0.28	-
113	Ui	42.64	2153	0.48	-
114	Kaurene	40.34	2051	-	0.79
115	Ui	43.51	2192	0.16	tr.
116	Ui	43.90	2210	0.15	0.58
117	Ui	44.45	2236	1.00	-
118	Ui	45.55	2288	0.20	-
119	Isopimarol	46.14	2316	0.11	-
120	Ui	51.61	2563	0.41	-
Total (%)				99.97	99.82

RT—retention time; RI—retention indices on VF-5 ms column relative to C7-C40 n-alkanes; Ui—unidentified compound with a marked mass spectrum; and tr—trace amounts.

**Table 3:** Description and characteristics of *S. scardica* plant material.

Material Designation	Name	Origin	Producer
SEO 1	*S. scardica* herb	Bulgaria	PPH Astron; Stary Henryków, Poland
SEO 2	*S. scardica* herb	Türkiye	Bakra Natura; Gdańsk, Poland

## Data Availability

The raw data supporting the conclusions of this article will be made available by the authors on request.

## Supplementary Materials

The following supporting information can be downloaded at: https://www.mdpi.com/article/10.3390/molecules31091515/s1, Figure S1 The structure of the major compounds in the *Sideritis scardica* essential oils.
