# Supplementary material for: Sideritis scardica Griseb. Essential Oil as Potential Antimicrobial Agents—A Study of Their Composition and Activity
Source: Molecules. 2026 May 2;31(9):1515. doi: 10.3390/molecules31091515 (PMC13164679; doi:10.3390/molecules31091515)
Supplement: Supplementary file 1 [file molecules-31-01515-s001.zip › molecules-4264130-supplementary.pdf]

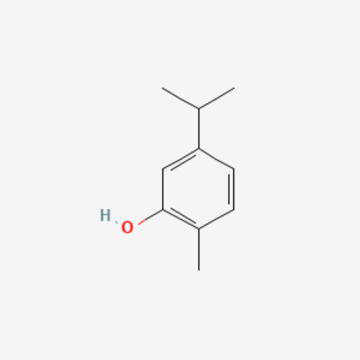

https://pubchem.ncbi.nlm.nih.gov/compound/Carvacrol

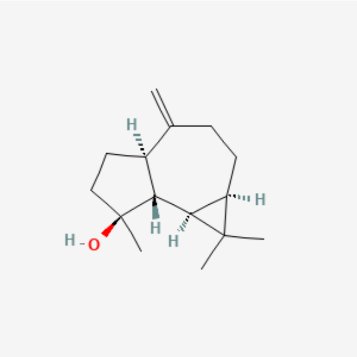

https://pubchem.ncbi.nlm.nih.gov/compound/Spathulenol

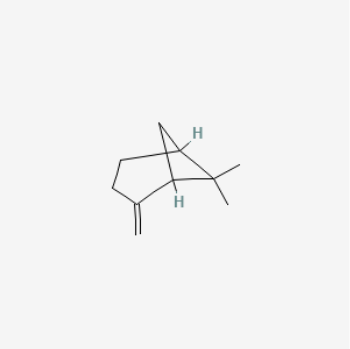

https://pubchem.ncbi.nlm.nih.gov/compound/Beta-Pinene

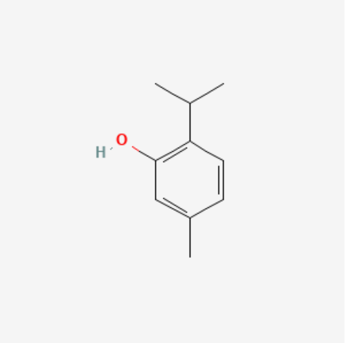

https://pubchem.ncbi.nlm.nih.gov/compound/Thymol

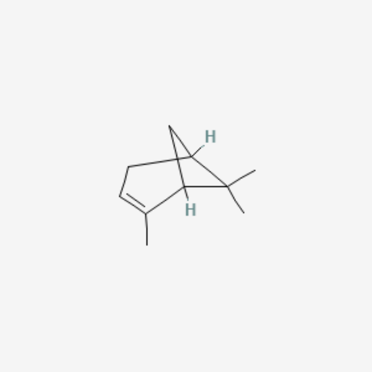

https://pubchem.ncbi.nlm.nih.gov/compound/alpha-Pinene

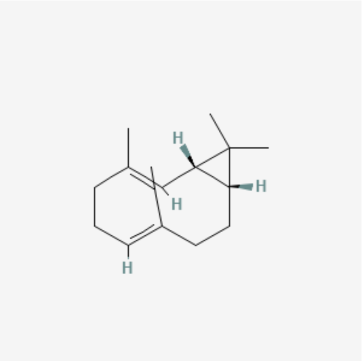

https://pubchem.ncbi.nlm.nih.gov/compound/13894537

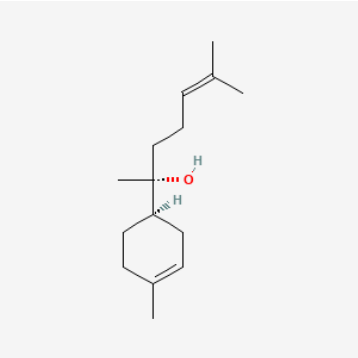

https://pubchem.ncbi.nlm.nih.gov/compound/Bisabolol

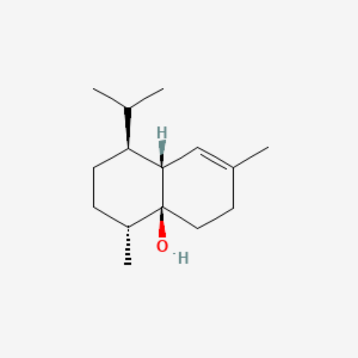

https://pubchem.ncbi.nlm.nih.gov/compound/12046149

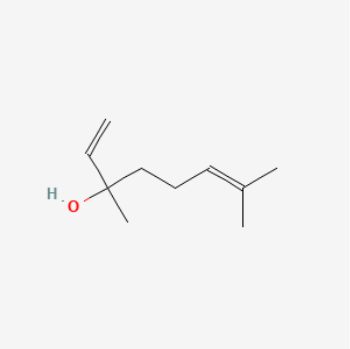

https://pubchem.ncbi.nlm.nih.gov/compound/Linalool

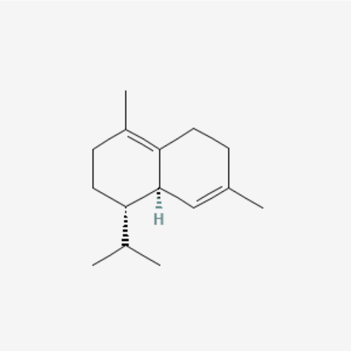

https://pubchem.ncbi.nlm.nih.gov/compound/delta-Cadinene

Figure S1. The structure of the major compounds in the *Sideritis scardica* essential oils.
